# Variability of a natural hydrocarbon seep and its connection to the ocean surface

**DOI:** 10.1038/s41598-020-68807-4

**Published:** 2020-07-28

**Authors:** Mahdi Razaz, Daniela Di Iorio, Binbin Wang, Samira Daneshgar Asl, Andreas M. Thurnherr

**Affiliations:** 1 Department of Marine Sciences, University of Georgia, Athens, GA USA; 2 Department of Civil & Environmental Engineering, University of Missouri, Columbia, MO USA; 3 Geography Department, University of California, Santa Barbara, CA USA; 4 Lamont Doherty Earth Observatory, Columbia University, Palisades, NY USA

**Keywords:** Environmental impact, Physical oceanography

## Abstract

Natural hydrocarbon seeps are ubiquitous along continental margins. Despite their significance, we lack a basic understanding of the long-term temporal variability of seep dynamics, including bubble size, rise velocity, composition, and upwelling and entrainment processes. The shortcoming makes it difficult to constrain the global estimates of oil and gas entering the marine environment. Here we report on a multi-method approach based on optical, acoustic, satellite remote sensing, and simulations, to connect the characteristics of a hydrocarbon seep in the Gulf of Mexico to its footprint on the sea surface. Using an in-situ camera, bubble dynamics at the source were measured every 6 h over 153 days and the integrated total hydrocarbon release volume was estimated as 53 m^3^. The vertical velocity was acoustically measured at 20 m above bed (mab) and found to be approximately 40% less than the dispersed-phase at the source, indicating that the measured values are reflecting the plume continuous-phase flow. Numerical simulations predict that the oily bubbles with diameters larger than 8 mm reach the surface with a small footprint, i.e. forming an oil slick origin, deflection of which with wind and surface current leads to the formation of an oil slick on the surface. Nineteen SAR images are used to estimate the oil seepage rate from GC600 for 2017 giving an average discharge of 14.4 cm^3^/s.

## Introduction

Natural hydrocarbon seeps have been reported from a broad range of oceanographic settings from the coast to the deep ocean over a wide variety of geological environments that affect the biosphere, the hydrosphere, and the atmosphere^1^. Beside the anthropogenic sources, hydrocarbon seeps are the single most important natural source of oil and methane (CH_4_) that enter the ocean^2^. Best estimates indicate that 3 to 48 Tg/y of geo-CH_4_ is released from marine seeps alone^3,4^. Recent global estimates of crude oil seepage range from 0.2 to 2 Mt with a best estimate of 0.6 Mt that accounts for 47% of the global estimate. The large uncertainties in estimates of hydrocarbons released from offshore marine sources are due to a wide range of factors including size, rise velocity, contamination by surfactants such as gas hydrates, composition, upwelling and entrainment effects, and interaction of gaseous bubbles and oil droplets with the local ocean environment that vary constantly with time.

To quantify the bubble/droplet size and rise velocity and the corresponding emission rate of hydrocarbons from the seafloor, investigators have primarily relied on snapshot acoustic^5–9^ or optical^10–13^ measurements. Based on these measurements, significant changes in hydrocarbon seepage and bubble venting have been inferred to occur over intervals of months to years^14^. Sufficiently long time series of seepage characteristics rarely exist to conclusively address the day-to-day variability of bubble size and rise velocity or the spatial changes in vent location over an extended period. Time variation in seep emissions is of particular interest. It implies variability in the local background levels against which pollution from human activities is measured, and is relevant at a global scale when seepage is scaled up to all continental margins^15,16^.

**Figure 1:**
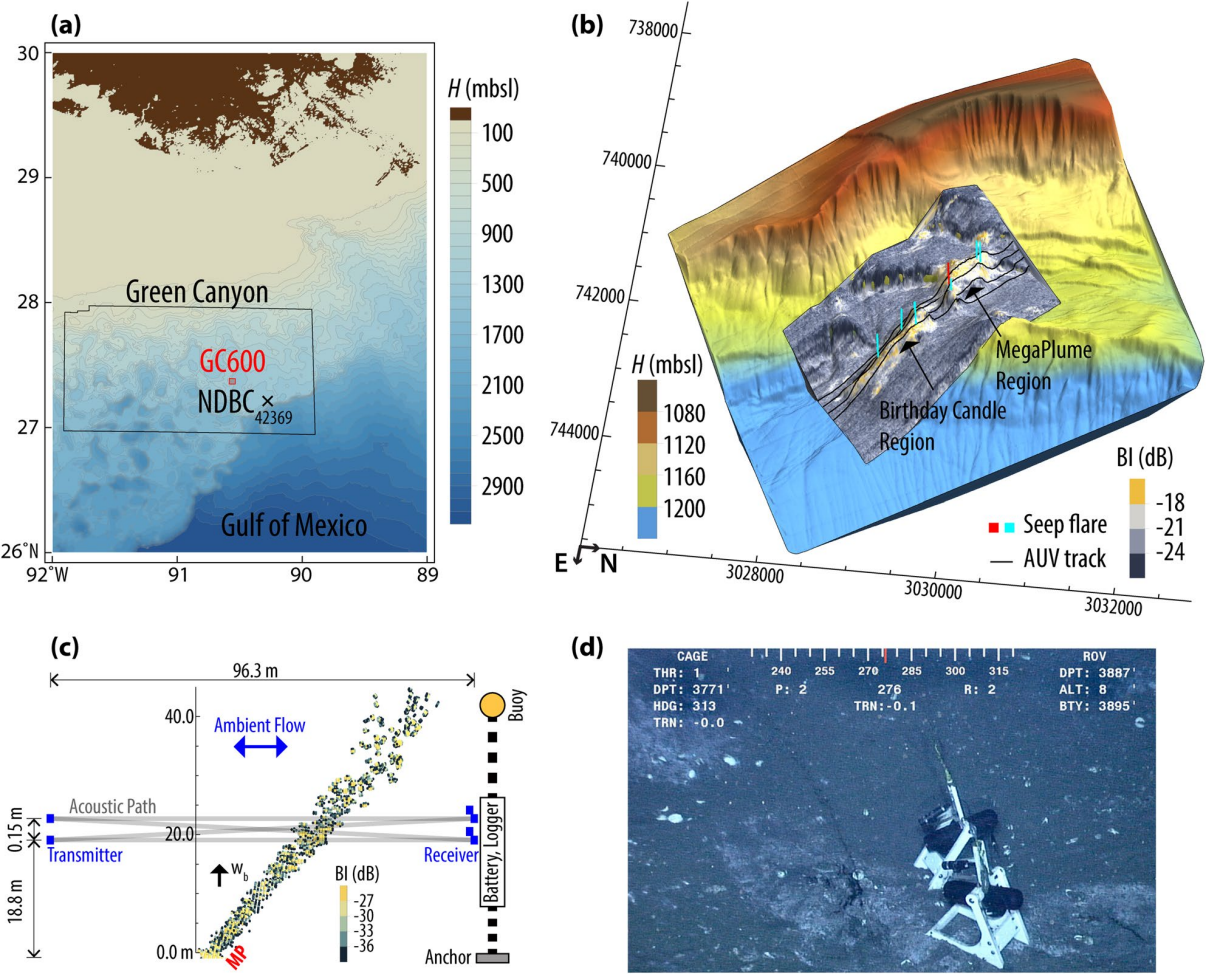
(**a**) Green Canyon (GC) lease block area together with the GC600 perimeter outlined in red, as designated by the Bureau of Ocean Energy Management (BOEM), superimposed on high resolution bathymetry of GoM from Becker, et al.^24^. Also shown is the National Oceanic and Atmospheric (NOAA)/National Data Buoy Center (NDBC) station 42369 (27° 12.4000 N, 90° 16.9667 W). (**b**) High-resolution (1 m) 3D view of a part of GC600 recreated with permission from Mitchell, et al.^25^. Superimposed is the area surveyed with a Kongsberg EM2040 multibeam sonar shaded with backscatter intensity. The bright areas indicate extensive methane-rich fluid or gas discharge leading to the formation of authigenic carbonates on the seafloor. Also shown are major seep flares identified from water column backscatter anomalies having intensities larger than − 40 dB; the Mega Plume flare is highlighted in red. Coordinates are in UTM15N/WGS84 datum. (**c**) Backscatter anomalies caused by bubbles released from Mega Plume as collected by the multibeam echosounder from 40 mab. Superimposed is the schematic deployment of the acoustic scintillation flow meter used to monitor the upwelling flow at 20 mab. The sketch is not in scale. (**d**) Photo snapshot taken by the ROV Millennium aboard MSV Connor Bordelon from the Mega Plume seep on Sept 3, 2017, with the two video time-lapse cameras visible in the image.

The Green Canyon 600 lease block (GC600) is located in the northern Gulf of Mexico (GoM, Fig. 1a). Satellite image records containing chronically visible surface oil slicks suggest it is one of the most prolific natural hydrocarbon seep sites in the region^17,18^. Geophysical studies affirm these findings and show abundant seep formations, including reservoirs, faults, and migration conduits found in seismic profiles^19,20^. The abundance of seeps has made this region an attractive site for studying different biological and physical aspects of natural methane and oil emission^20–23^. Here, we report on a comprehensive observational and modeling approach based on optics for time-lapse video measurements of bubble dynamics and size distribution, acoustics for detecting plume-induced upwelling flow, numerical modeling to predict the migration path of hydrocarbons in the water column, and satellite remote-sensing to quantify the oil discharge from its footprint on the sea surface. As a result, this paper describes an unprecedented study that explores the dynamics of a submarine seep and its interaction with the surrounding environment from the seafloor to the sea surface.

## Measurement program

To identify a natural hydrocarbon seep with a visible oil slick on the surface we surveyed the seepage area (1,600 × 2,200 m^2^) within the GC600 block using an autonomous underwater vehicle (AUV) equipped with a multibeam sonar (see “Methods”). Rising bubbles through the water column scatter acoustic energy effectively, instigating flare-like structures in hydroacoustic data. Figure 1b demonstrates some of these flares caused by gas bubbles nominally larger than 0.2 mm in diameter, which were utilized to localize their seabed source. The seep flares are superimposed on a 3D view of the bathymetry obtained from the multibeam data; shading corresponds to the backscatter intensities. The bathymetry map demonstrates a salt-supported ridge trending NW–SE with two distinctive areas of high-reflectance seafloor. While the observed hydrocarbon seep locations lack distinct morphology, they are highly correlated with patches of high seafloor reflectivity that may be associated with methane-derived carbonate slabs and crusts^26,27^.

Diving with the remotely operated underwater vehicle (ROV) Comanche aboard OSV Ocean Project enabled us to visit some of the hydrocarbon plumes directly. Figure 1c,d respectively display the acoustic image and a photo snapshot of the hydrocarbon seep, Mega Plume, which was selected for further measurements. The water depth at the Mega Plume, 27° 22.1915′ N, 90° 34.4580′ W, is approximately 1,180 m. It is about 10 m away from the vents described by Johansen et al.^11^ and Wang et al.^10^; these seep locations were not active during our visit, suggesting a high spatial variability of seeps in the Mega Plume region.

## Hydrocarbon source characteristics

**Figure 2:**
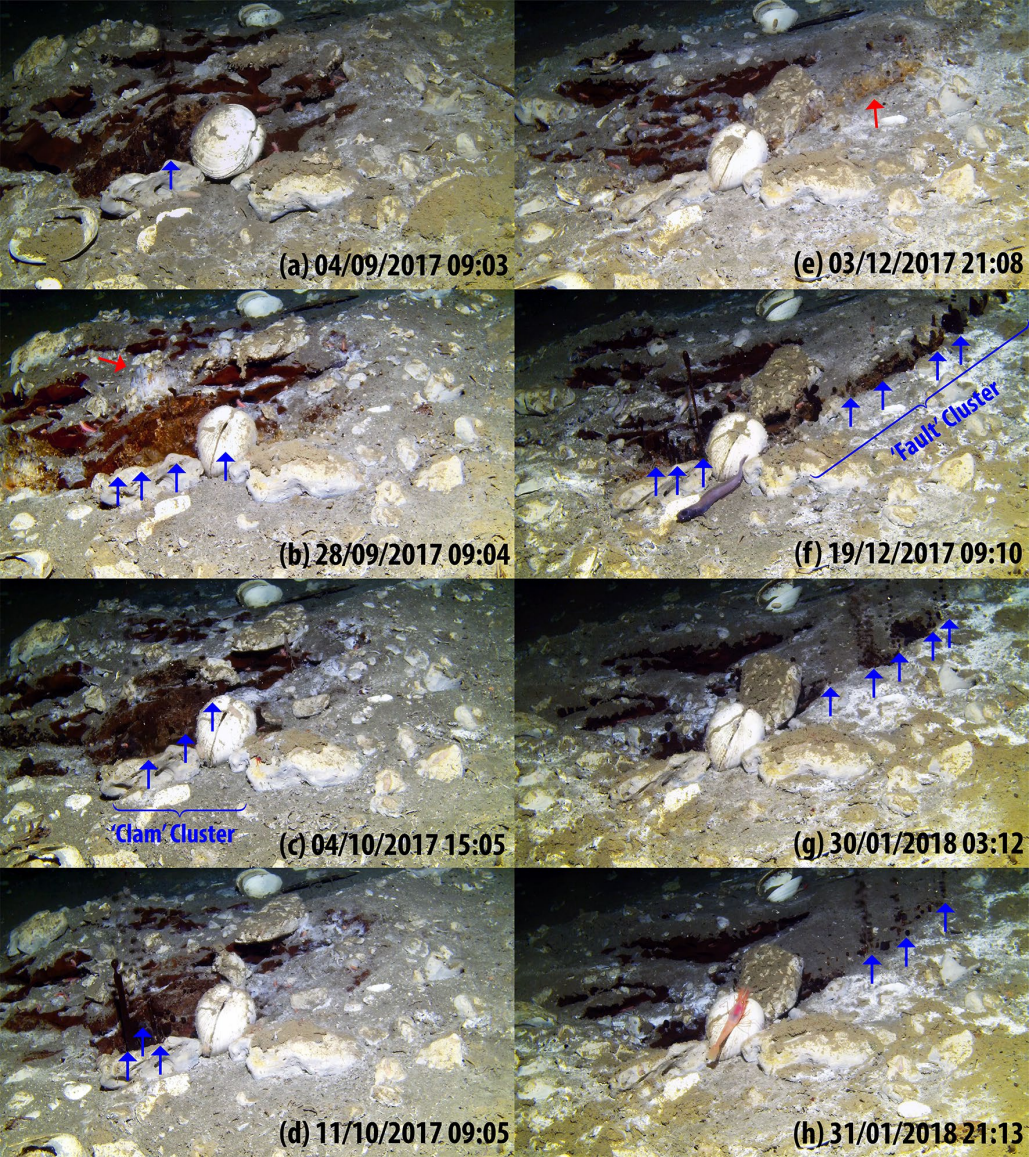
Image snapshots of the significant events captured by the VTLC-B through the deployment period of Sept 3, 2017–Feb 2, 2018. The seep site was occupied by at least one live vesicomyid clam and rubble comprising carbonate nodules and clam shells. The left column (**a**–**d**) exhibits the snapshots associated with the Clam Cluster and the right column (**e**–**h**) with the Fault Cluster. Blue arrows denote the individual vents and gas hydrate outcrops are marked with red arrows, where visible.

To explore the time–space variability of the seep at the seafloor, we positioned two video time-lapse cameras (VTLCs)^11^ close to the seep cluster (Fig. 1d, see “Methods”). A short video made from all the last 10 frames (1/3 s) of each of 568 video bursts recorded by the VTLC-B (see Video 1 in the supplemental document) exhibits day-to-day fine-scale spatial and temporal variability of the source over the campaign period. The records indicate that the seafloor and the seepage were very dynamic on daily to monthly time scales. Initially—from Sept to late Nov 2017—gas bubbles, oil droplets, and/or a mixture of the two were released from the edge of an exposed gas hydrate deposit identified as the ‘Clam Cluster’ in Fig. 2a–d. The dense cluster included one to three vents shifting intermittently over an approximately 10 cm long region. The emission from this vent cluster fully ceased on Dec 2, 2017, for the first time; concurrently, small patches of oil-stained methane hydrate outcrop started to emerge along what appeared to be a new crack or fault line on the seafloor (marked as the ‘Fault Cluster’ on Fig. 2e–h). Within about 2 days, chimney-like tubes of oil formed along both clusters and started releasing oily bubbles at low frequency. On Dec 14, both clusters were completely active emanating oily bubbles. Along approximately 20 cm of the Fault Cluster captured by the VTLC-B, up to 9 individual vents could be identified. The emission from the Clam Cluster ceased completely on Dec 28, 2017, for the rest of the campaign. Visual inspection revealed that while the composition of the bubbles emanating from different vents in the Clam Cluster were changing uniformly, the multiple vents along the Fault Cluster released either purely gaseous, oily bubbles or a combination of both, simultaneously. Another interesting feature to note from the time-lapse video is that the seafloor elevation started to change over the entire period – the evident subsidence may be associated with the breakdown of gas hydrate.

**Figure 3:**
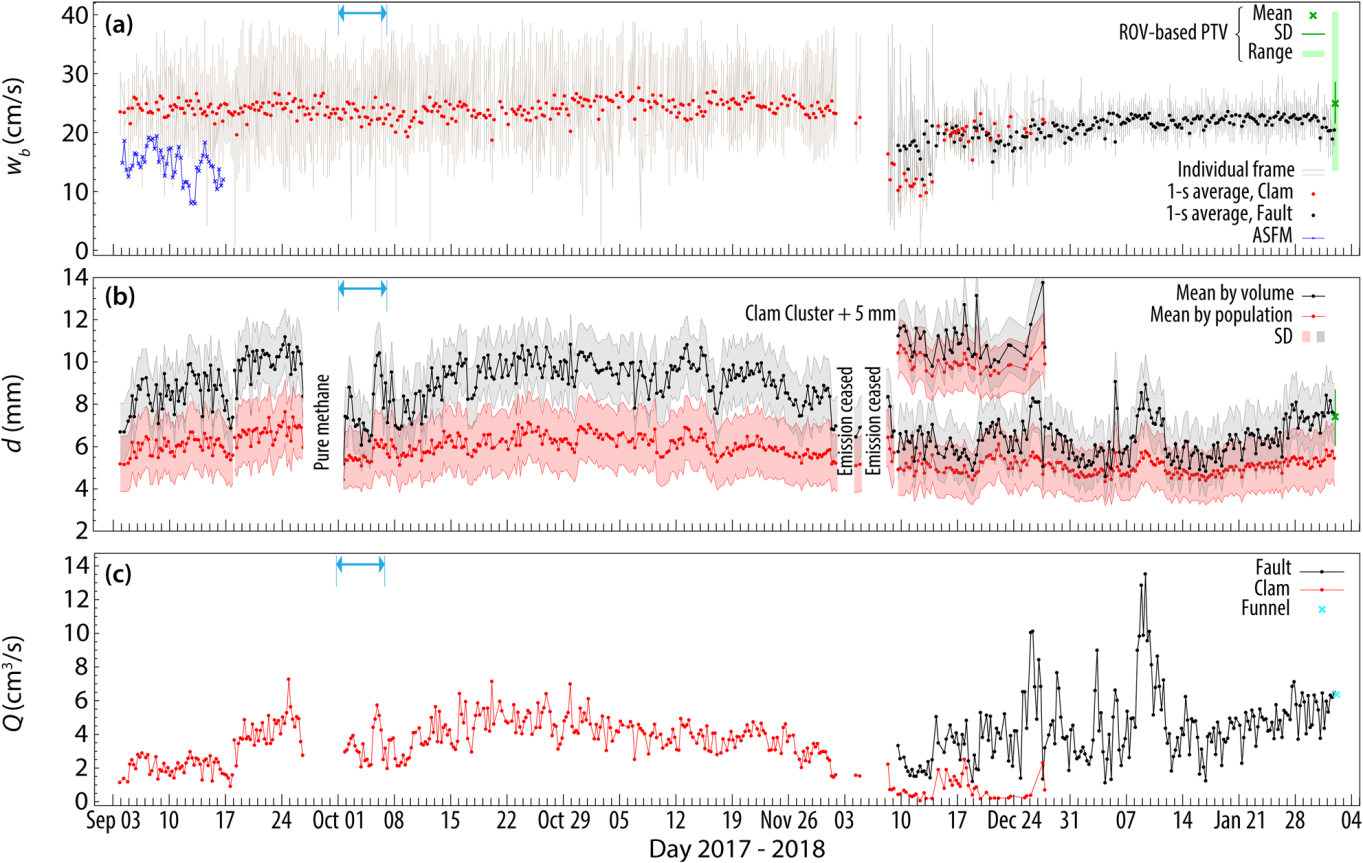
(**a**) Bubble rise velocity at the source obtained from the VTLC-B records averaged over each frame (regardless of the size, thin gray lines) and over 1 s (disks) for the Clam and Fault Clusters with comparison to the ROV-based measurement, ${{\stackrel{-}{w}}_{b}}_{\text{PTV}}$ (green). Also shown is the upwelling velocity measured by the ASFM during the first two weeks of the campaign at 20 mab (blue). (**b**) Mean bubble diameter based on population and volume weights, with ROV-based measurement ${{\stackrel{-}{d}}_{v}}_{\text{PTV}}$ superimposed (green). The outlines denote a confidence interval of ± 1 SD. The Clam Cluster data in Dec is shifted 5 mm upward to prevent clutter. (**c**) Source emission rate from the individual bubble size, rise velocity, and frame rate of the VTLC. The synoptic physical collection of hydrocarbons into an ROV-held funnel is also shown (cyan). The horizontal blue arrow denotes the time interval through which transport and fate of oil-coated methane bubbles in the water column are simulated by the TAMOC-SBM model.

**Figure 4:**
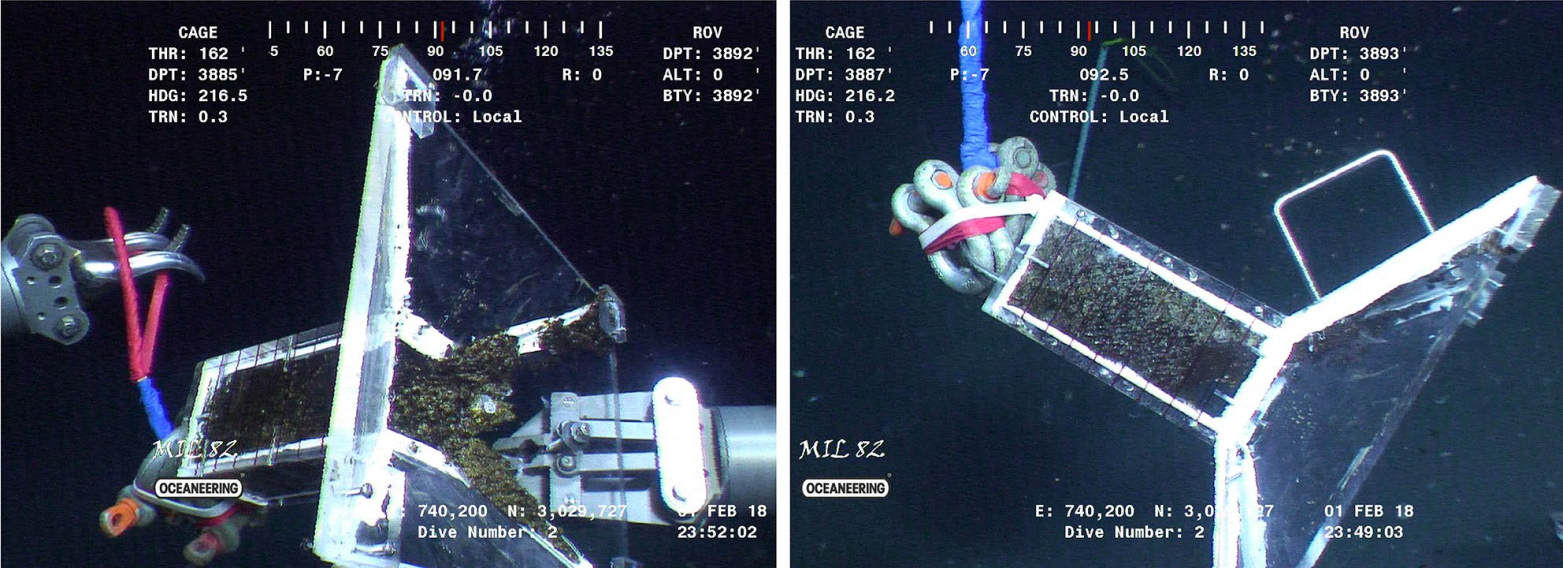
Sample pictures of the custom-made funnel used to measure the instantaneous flux rate of the Mega Plume. The funnel has a 2000 cm^3^ compartment divided into 8 visible parts. The ROV Millennium manipulator (right image) shows the solid nature of the oil and methane hydrate mixture.

The VTLC capability of autonomously recording videos over a long period comes at the cost of reduced frame rate and illumination compared to laboratory^28^ and ROV-based techniques^10^. Therefore, we developed a sequence of novel semi-supervised algorithms based on standard Mathematica^®^ and ImageJ^®^ routines to resolve the rise velocity, *w*_*b*_, and size, *d*, of individual bubbles, details of which can be found in Razaz et al.^29^. Utilizing these algorithms, 1 s of each video burst was processed. Figure 3 demonstrates the VTLC-B data together with the complementary ROV-based particle tracking velocimetry (PTV) results (see “Methods”) and the synoptic physical collections of hydrocarbons into an ROV-held funnel (see “Methods”, Fig. 4).

At the Clam Cluster, the average bubble rise velocity (Fig. 3a) with a mean bubble diameter of ${\left.{\stackrel{-}{d}}_{v}\right|}_{\text{Clam}}=9.0\pm 1.4$ mm was estimated to be ${\left.{\stackrel{-}{w}}_{{b}_{\text{VTLC}}}\right|}_{\text{Clam}}=24.1\pm 3.6$ cm/s. The smaller bubbles released from the Fault Cluster with ${\left.{\stackrel{-}{d}}_{v}\right|}_{\text{Fault}}=6.3\pm 1.3$ mm ascended at a slightly lower average velocity of ${\left.{\stackrel{-}{w}}_{{b}_{\text{VTLC}}}\right|}_{\text{Fault}}=17.5\pm 4.5$ cm/s. The subscript $v$ in these measurements denotes the weighting of the probability density function (PDF) by volume of bubbles (black curve in Fig. 3b) and the overbar stands for averaging over time. Also shown are the bubble sizes determined by fitting a log-normal distribution to the bubble size PDFs calculated for each 1 s sequence and bubble volume weighted PDFs. The VTLC bubble size and rise velocity averaged over the last 24 h are also compared to the ROV-based PTV results and are respectively, 5% and 20% lower, but are within the range of the complementary measurements. In general, our findings are consistent with previous works^7,10,13,30^ that reported velocities less than 30 cm/s for bubbles released within the hydrate stability zone having *d* < 26 mm.

Figure 3c demonstrates the emission rates, *Q*_VTLC_, calculated using the bubble size, rise velocity, and frame rate of the VTLC-B. There is outstanding compliance (2% difference) between the ${\stackrel{-}{Q}}_{\text{VTLC}}=6.5$ cm^3^/s measured 2.5 h before the synoptic physical collection of hydrocarbons with an ROV-held funnel (Fig. 4), ${Q}_{\text{FNL}}=6.4$ cm^3^/s. Of more importance is the significant difference between the volume of hydrocarbon estimates from long-term VTLC records and snapshot funnel measurements. That is, integrating the *Q*_VTLC_ values^29^ over the entire period of our campaign yields an emission volume of 51 m^3^. Had we used the constant ${Q}_{\text{FNL}}$ instead, the total emission would be overestimated by 63% (83 m^3^) over the same period. This large divergence signals the uncertainty that might be involved in annual estimates of methane released into the hydrosphere from ROV-based synoptic measurements. The difference between the seepage characteristics of the two clusters is summarized in Table 1 which quantifies the bubble dynamics and hydrocarbon discharge. The differences may be related to a wide range of factors including sediment loading, migration pathways, hydrate formation, permeability, and porosity^31^.

**Table 1:** Mega Plume summary of the mean (μ) and standard deviation (σ) for bubble size (*d*), rise velocity (*w*_*b*_) and discharge (*Q)*.

Cluster	*d* (mm)				$w_b$ (cm/s)		*Q* (cm^3^/s)	
	μ	σ	μ*v*	σ*v*	μ	σ	μ	σ
Clam	6.1	1.4	9.0	1.4	24.1	3.6	3.8	1.2
Fault	5.0	1.3	6.3	1.3	17.5	4.5	4.3	1.8

The data from Clam Cluster are averaged over Sep 3–Nov 9, 2017, and for the Fault Cluster over the available data.

**Figure 5:**
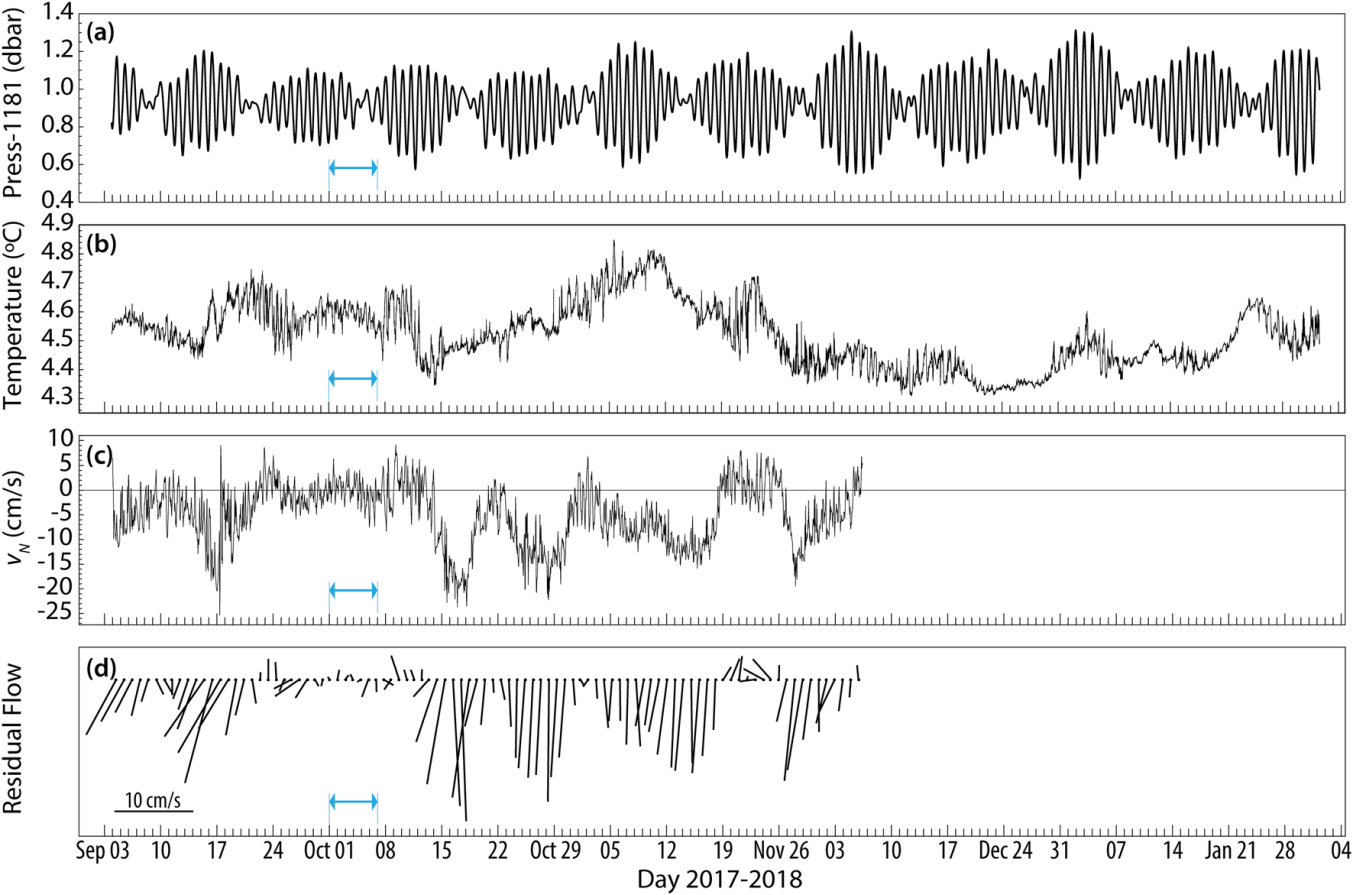
(**a**) Tide-induced pressure variability at 4 mab and (**b**) temperature variability at 24 mab both recorded with MicroCAT conductivity-temperature-depth sensors approximately 50 m away from the seep source. (**c**) Hourly-averaged north/south current component and (**d**) daily-average residual flow magnitude and direction obtained by passing the hourly data, taken at 20.6 mab from the 600 kHz acoustic Doppler current profiler (ADCP), through a 36-h low-pass filter.

Many processes can be postulated to explain short-term (days to weeks) variations in seepage rate including variations in bubble characteristics (size, shape, and composition), properties of gas–liquid systems (density, viscosity, surface tension, density difference between gas and liquid, oil coating, and formation of methane-hydrate) and oceanographic conditions (temperature, pressure, and crossflow velocity)^29^. Sufficiently long time series of seepage characteristics together with the background hydrographic conditions in deep waters rarely exist to conclusively identify the effects of each of the postulated mechanisms. Although variations in gas seepage due to tidal forcing have been documented in some shallow sites (< 70 m)^32,33^, comparing the depth fluctuations induced by tides in our survey site (Fig. 5a) with the variability of seepage characteristics demonstrated in Fig. 3, does not reveal any apparent correlation, presumably because of the relatively trivial tidal depth fluctuations (Fig. 5a) compared to the water depth. Furthermore, the bubble rise velocity (Fig. 3a) and mean size (Fig. 3b), are not in phase with depth or dominant ambient current fluctuations, *V*_*N*_, induced by tides or residual dynamics (Fig. 5c,d). The subscript *N* denotes currents towards north/south. The background temperature (Fig. 5b) recorded at 24 mab varied over a narrow range of 4.3–4.8 °C during the observation period. Although a few tenths of a degree increase can induce dissociation in gas hydrates^34^, the ambient pressure and temperature here are well within the methane hydrate stability zone^35^. On longer time scales, the seep characteristics may be affected by pressurization of oil and gas in reservoirs, and differential loading of sedimentary layers^36^. Besides, gas hydrate deposits and subsurface oil and gas pockets potentially impinge hydrocarbon releases and could influence bubble size and gas:oil ratios.

### Plume-induced upwelling velocity at 20 m above the seafloor

We measured the vertical velocity of the plume-induced flow using an acoustic scintillation flow meter (ASFM)^37–39^ (see “Methods”) over two weeks, Sept 3–17, 2017. In précis, the measurement concept relies upon the scattering field to advect across spatially separated acoustic paths and requires persistence of those structures to obtain useful coherence in the received signals at the receivers. As indicated by visual and acoustic imaging (Fig. 1c), the bubble plume expands with height above the bed by entraining fluid and by dispersive mechanisms; comparing the ASFM, ${\stackrel{-}{w}}_{\text{ASFM}}=12.9\pm 2.9$ cm/s, with the VTLC results averaged over the same period suggests the increase in mass flux led to approximately 40% lower upward velocities at 20 mab compared to the source. Given the ASFM configuration at GC600 (see “Methods”), the ASFM was sensitive to turbulence length scales of approximately 0.6 m that lie within the inertial subrange of fine-scale turbulence^40^. One implication of this scale is that the ASFM treats the water (continuous-phase) and bubbles (dispersed-phase) as a mixed turbulent medium advected by a vertical velocity that is weighted towards the center of the acoustic path. Therefore, we hypothesize that the measured values are reflecting the vertical velocity of the plume continuous-phase (upwelling velocity) and do not include the dispersed-phase. It is also noted that there is no evident correlation between the upwelling flow variability and the ambient currents induced by the tide or those occurring at lower frequencies (Fig. 5).

### Transport of hydrocarbons in the water column

**Figure 6:**
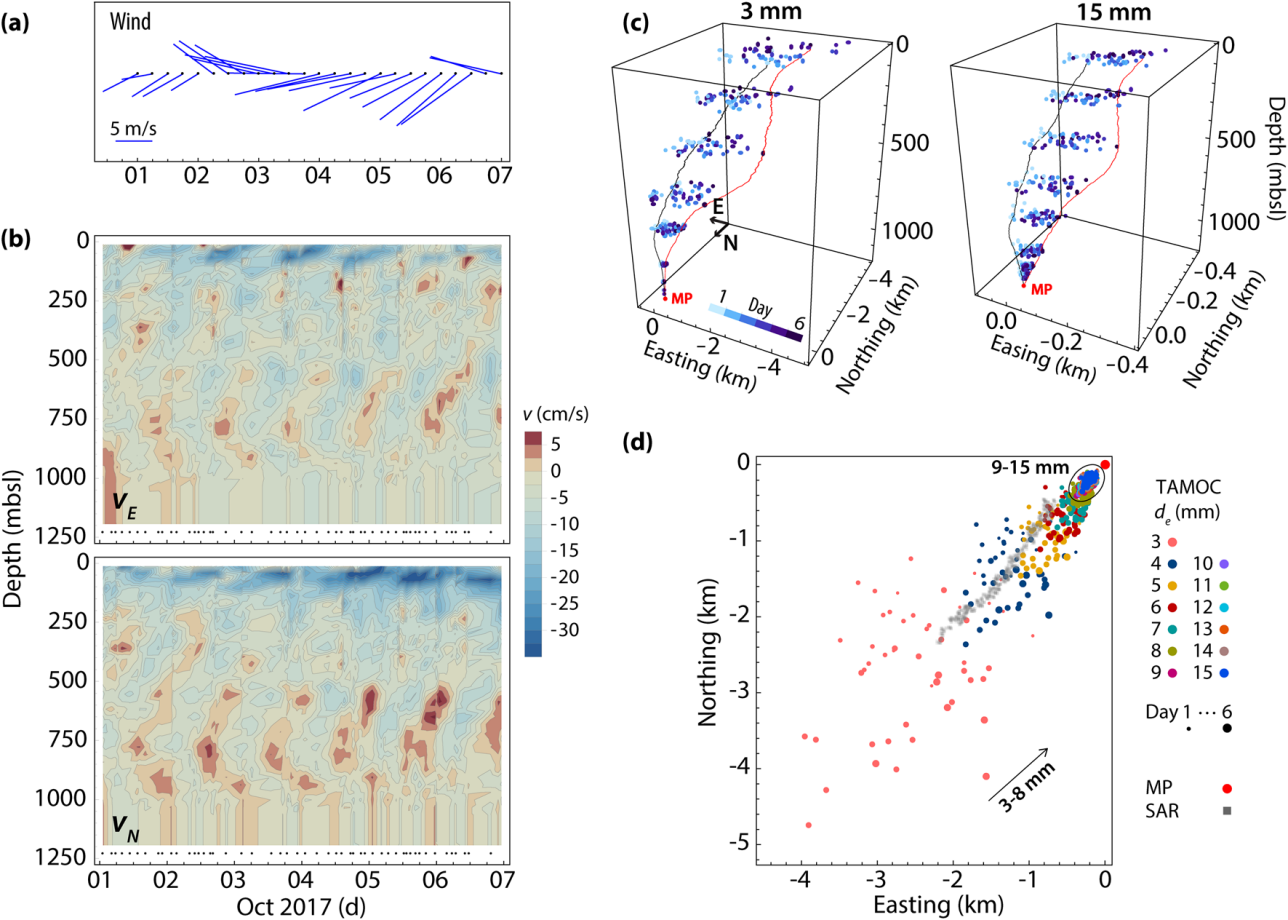
TAMOC-SBM simulations. (**a**) The 10 m wind data over Mega Plume from ERA-Interim Archive provided by the European Centre for Medium-Range Weather Forecasts (ECMWF)^43^ over a 0.125° × 0.125° grid every 6 h. (**b**) Ambient current registered by three ADCPs from the National Oceanic and Atmospheric Administration (NOAA)’s National Data Buoy Center (NDBC) station 42,369 located at 27° 12.4000′ N, 90° 16.9667′ W, approximately 80 km from Mega Plume. The average of the last four good bins is replaced with the missing bins to the bottom when no records were available near the seafloor. (**c**) The migration path of oily bubbles from the seafloor (1,180 m depth) to the sea surface over 6 days for bubbles with *d* = 3 mm and 15 mm. The two polylines are samples of the trajectory of bubbles released on Oct 4 01:19 (black) and Oct 6 19:09 (red). (**d**) Surfacing footprint of bubbles over Oct 1–6, 2017, obtained from TAMOC-SBM for the range of bubble sizes simulated. For comparison, the oil slick delineated from a SAR image captured by the Sentinel-1A satellite on Oct 1, 2017, is also shown. Eastings and northings are referenced to Mega Plume (MP) shown as a red dot.

We used the single bubble model (SBM) of the Texas A&M Oilspill Calculator (TAMOC)^41^ to track the trajectory and the evolution of bubble size, mass, and composition as a function of depth, which provides information on the final surfacing characteristics for these bubbles. The module takes into account background stratification, advection by ambient currents (see “Methods”, Fig. 6b), dissolution, and heat transfer. The bubbles were assumed to be gaseous coated with oil in our simulations with sizes ranging between 1 and 15 mm (equivalent spherical diameter with a stride of 1 mm) at the source. For further details see “Methods”. The simulation spanned the period Oct 1–6, 2017, when a synthetic aperture radar (SAR) image was available for comparison.

The simulations imply that depending on the bubble size and currents, bubbles take substantially different migration paths, which subsequently affect the transit time of the bubble in the water column. Figure 6c illustrates the difference between two representative bubble trajectories having sizes *d* = 3 mm and 15 mm and their orbital path with time in the water column as a result of diurnal tides and inertial oscillations at approximately the same frequency. The average transit time of bubbles with *d* > 8 mm at the source is approximately 40% of that computed for bubbles with *d* = 4–8 mm. An effect of the decrease in transit-time is a reduction in the size of the surfacing footprint, evident in Fig. 6d, since there is less time for dispersal by turbulence and currents^42^. Also, comparing the surface outbreak of the bubbles with different sizes with the oil slick delineated from the SAR image captured by the Sentinel-1A satellite on Oct 1, suggests that only larger bubbles contribute to the formation of the oil slick origin (OSO)^18^. The surfaced oil is then elongated as a film that drifts away with surface currents and wind (Fig. 6a)^42^. Here, it is not possible to separate the effects of the prevailing wind and near-surface current on the general shape of the oil slick as both vectors were roughly aligned to the southwest.

### Surface footprint of oil seepage

**Figure 7:**
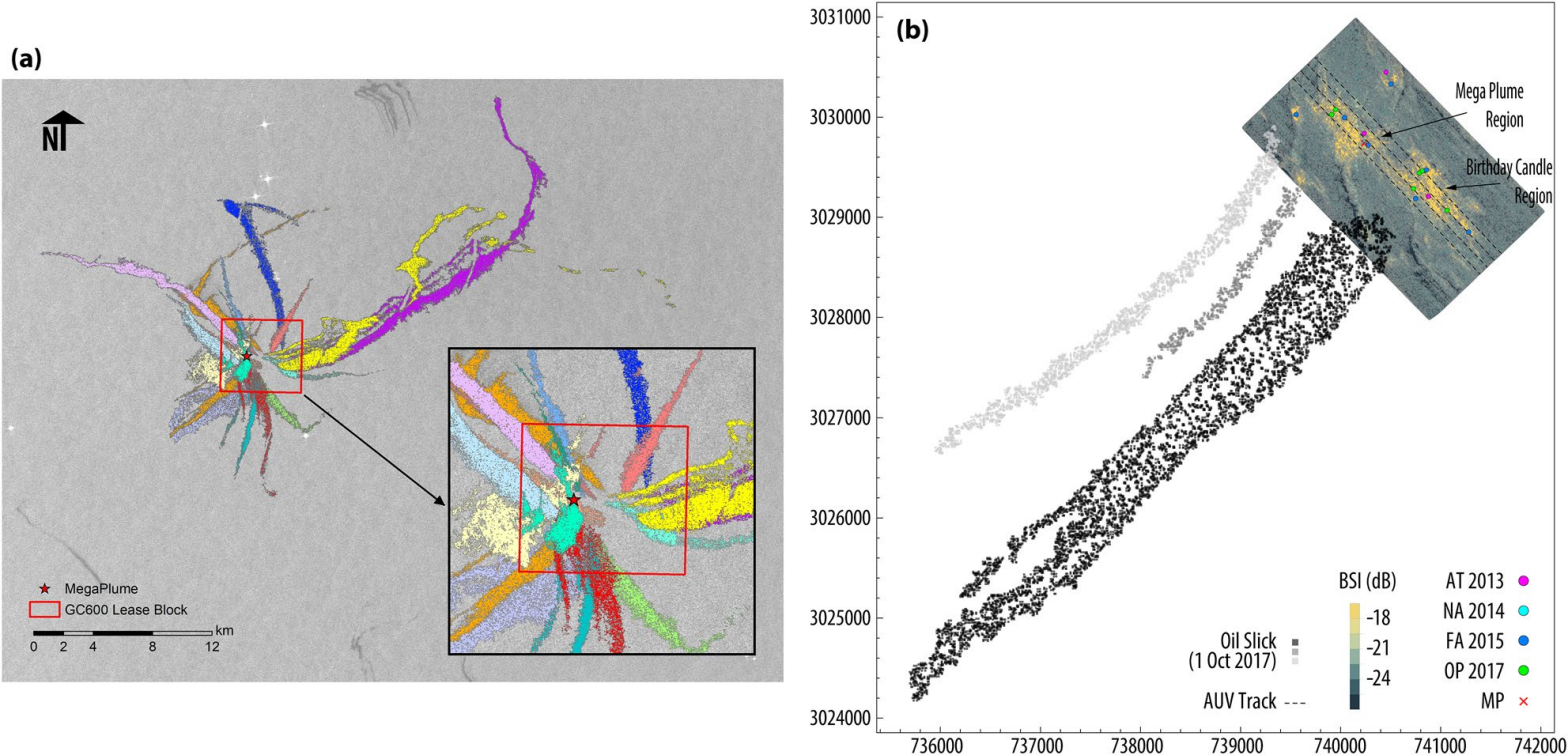
(**a**) The oil slicks originating from the Mega Plume region moving away from sources in the GC600 lease block (red box) in a continuous, wind- and current-driven orbit. Colors denote individual SAR images captured between February and December 2017. (**b**) The bathymetry map shaded with acoustic backscatter intensity obtained from the multibeam echosounder on Jun 18–19, 2017. Superimposed are seeps detected in the region from various cruises (2013 R/V Atlantis; 2014 E/V Nautilus; 2015 Fugro Americas; 2017 Ocean Project). Also shown are three oil slicks captured by the Sentinel-1A satellite on Oct 1, 2017, and corresponds to the time of the TAMOC modeling. Coordinates are in UTM15N/WGS84 datum.

To estimate the hydrocarbon flux from sources on the seafloor using the oil slick properties on the surface, we used 19 SAR images collected over GC600 and its surrounding region between Feb 2017 and Dec 2017. In these SAR images, the wind condition was favorable for the detection of oil slicks. A semi-automated image processing routine, the Texture Classifying Neural Network Algorithm (TCNNA)^44,45^, was employed to delineate the oil slicks shown in Fig. 7a. Based on the TCNNA results, the average surface residence-time is computed to be *t*_*R*_ = 8.6 h, and the average oil slick surface area is *Ā*= 4.45×106 m^2^ over the GC600 domain, taking into account the wind and surface current conditions (see “Methods”). Choosing a conservative and uniform oil film thickness of 0.1 µm following the published standards^46^, the oil seepage on the seafloor for the GC600 domain is calculated to be *Q*_SAR_ =14.4 cm^3^/s.

While processing the SAR images, the deflection of the OSO from the vent is approximated by a linear relation with water depth only^47^ and for GC600, the deflection distance can be as high as 2.3 km^18^. Considering the geographically dense seeps in this region, this coarse estimation degrades our ability to identify the venting source of oil slicks accurately. Figure 7b exhibits three distinct oil slicks on Oct 1, 2017, the middle of which is what we denote as coming from Mega Plume with a surface offset of approximately 1 km from the source as shown also in Fig. 6d; without extra information from the seabed, it is not possible to distinguish sources that fall within less than 2 km from each other. This outlines the value of independent measurements of the transport of oil/gas bubbles through the water column for identifying the surface footprint. If we consider the SAR image denoted in Fig. 3d as coming from the Mega Plume, given a surface residence time of 8.6 h, the discharge is estimated ${Q}_{{\text{SAR}}-{\text{MP}}}=2.4$ cm^3^/s, which compares favorably to our seep measurement of ${Q}_{\text{VTLC}}=3.0$ cm^3^/s for Oct 2, 2017.

## Methods

The extensive field survey carried out in 2017 in GC600, GoM, consisted of video observations of seep source physical conditions, acoustic Doppler measurements of near-bottom horizontal flows, acoustic forward scatter measurements of the vertical flow, and acoustic backscatter measurements of the water column and seafloor. Full water column horizontal currents were obtained from the nearest NDBC station 42369. These data sets were used to model the bubble rise through the water column for comparison to sea surface oil slick observations obtained from the Sentinel-1A SAR satellite. Each of these data sets is described below.

### Video-based observations

#### Video time-lapse camera (VTLC)

Two VTLCs were positioned at approximately 60 and 90 cm from the seep by the ROV Maxx aboard MSV Ocean Intervention II on Sept 3, 2017 (Fig. 1d). VTLC-A recorded 10 s of data every 3 h during Sept 3–28, 2017, and VTLC-B logged 15 s of data every 6 h over the period of Sept 3, 2017–Feb 2, 2018; a video of the 153-day deployment is provided in supplementary data, Video 1.

#### ROV-based particle tracking velocimetry (PTV)

A 10-min long video of the seep at the source was recorded using the Ocean ProHD TV camera (1080i/59.4) on the ROV Millennium during the recovery cruise on Feb 2, 2018. To surmount the inherent parallax error associated with a single-camera system, a screen with size scale markings was placed behind the seep. Following the PTV methods described in Wang and Socolofsky^48^, 350 s of the video was processed to determine the size and rise velocity of individual bubbles for comparison to the VTLC measurements.

#### Synoptic physical collection of hydrocarbons

A custom-made funnel shown in Fig. 4 was used to measure the emission rate over a period of about 5 min—the time it took to fill the funnel volume. The funnel volume compartment was drawn with 8 visible sections, each indicating 250 cm^3^. Using the ROV Millennium arm, the funnel was kept on top of the seep about 0.15 mab so that no hydrocarbons could escape the funnel while no disturbance was made to the loosely deposited sediment. It was not possible to repeat the sampling more than once since the compartment was filled with a solid mixture of methane hydrate and oil (Fig. 4).

### Acoustic observations

#### Multibeam echosounder

The primary survey sensor on the AUV aboard the Oceaneering vessel Ocean Project includes a Kongsberg EM2040 200 kHz multibeam sonar. An acoustically aided inertial navigation system consisting of an acoustic Doppler velocity log and an ultra-short baseline (USBL) positioning system on the survey vessel was used for accurate localization of the AUV. On Jun 18–19, 2017, we used the AUV to perform a systematic survey to identify natural hydrocarbon seeps in the seepage area of GC600. Throughout the survey, the AUV maintained an altitude of approximately 40 m with a speed less than 1.5 knots while cruising along 15 primary track lines spaced 100 m apart (Fig. 1b), and 3 tie lines spaced at 900 m. The water column and seafloor acoustic backscatter data were collected using 256 beams over a 140° wide swath (approximately 220 m on the seafloor in equidistance mode) at 4 Hz sampling frequency. High-resolution bottom bathymetry was also obtained from the bottom backscatter data.

#### Acoustic scintillation flow meter (ASFM)

An abundance of reports on the scintillation method to monitor horizontal and vertical flow is available in the acoustic and oceanographic literature^37–39,49,50^. The scintillation method in its simplest configuration consists of two transmitters emitting acoustic pulses in succession at a fast repetition rate. Each pulse is then received at two spatially spaced receivers to resolve turbulent fluctuations focused on the Fresnel scale, $\sqrt{\lambda L}$, where λ is the acoustic wavelength and *L* is the acoustic path length. A crucial requirement for the scintillation method is that there must be sufficient coherence between signals detected at the two receivers. Furthermore, the acoustic scattering must remain weak for reliable amplitude fluctuations^40^. From the time-lagged spatial coherence pattern, the path-averaged current orthogonal to the transmission path is obtained without requiring any geometric correction.

The ASFM, deployed in GC600, has been used in a variety of other deep-sea environments to monitor Mediterranean flow into the Black Sea and hydrothermal plume dynamics^38,49,50^. As depicted in Fig. 1c, using moorings carefully positioned 96.3 m apart, the transmitter and receiver arrays were deployed such that the acoustic propagation paths intersect the bubble stream at ~ 20 mab. The 100 μs transmit pulses with a central frequency of 307 kHz were emitted sequentially with 25 ms separation at 10 Hz sampling frequency. The instrument logged data in burst sampling mode, 15 min every hour. Application of toroidal transducers with a 10° beam width ensures that the sound is propagated through the bubble stream, regardless of where the stream bends.

#### Acoustic Doppler current profiler (ADCP)

The background flow in the near-bottom region was measured using a 600 kHz TRDI Workhorse ADCP mounted on a bottom tripod. The tripod was positioned 68 m from the Mega Plume seep cluster to avoid acoustic interference with the ASFM or with the bubble stream. The range cell size was set to 2 m with the first bin at 2.64 mab with zero blanking distance. The radial beam velocities were recorded in burst mode over the period of Sept 3–Dec 7, 2017, without any ensemble averaging with 912 s (at 0.76 s sampling interval) of data every hour. The radial beam velocities were transformed into East-North-Up (ENU) coordinates using the weighted least squares approach described by Gilcoto et al.^51^. The magnetic declination at the survey site (0.24° W) obtained from the ‘Magnetic Field Calculator’ readily available on the NOAA website. The magnetic declination is negligible compared to the accuracy of the ADCP compass (± 2°). The hourly averaged dominant north/south flow is exhibited in Fig. 5.

The primary sources of real-time current profile data for the NOAA-NDBC program in the GoM are the ADCPs suspended from or attached to oil production platforms and drilling rigs. The current profilers may look downward (from near the surface or at mid-depth), upward (from a bottom mount or at mid-depth), or horizontally (from near the surface). The raw data and the decoded data with quality control flags are available on the NDBC website. Here we used the archived data from the NDBC station 42369 located at 27° 12.4000′ N, 90°, 16.9667′ W with 1,371.9 m depth (Fig. 1a). Almost 80 km away from the Mega Plume, this is the nearest NDBC station to our seep site with consistently high-quality data throughout the water column. At this station, three ADCPs positioned at approximate depths of 12 (down-looking), 450 (up-looking), and 450 m (down-looking) covered the range 16–132, 148–420, 470–1,050 m respectively sampling every 20 min; results are summarized in Fig. 6b.

### Physical properties of seawater

Ancillary physical water column data including conductivity, temperature, and depth (CTD) profiles were gathered from an array of SeaBird SM37SB MicroCATs attached to the ASFM receiver mooring. The MicroCATs logged data every 90 s throughout Sept 4–Feb 3, 2017. Temperature variability recorded at 24 mab is given in Fig. 5.

### Simulating bubble dynamics with TAMOC

The composition of bubble contents in TAMOC-SBM was taken from Wang, et al.^10^, who collected samples of the gas bubbles released from a seep located at 27° 22.1954′ N, 90° 34.2624′ W in the Mega Plume region. Their work indicated that methane with 87.63% mol is the major constituent of the bubble contents. By adding other minor gaseous constituents including ethane, propane, nitrogen, carbon dioxide, and other hydrocarbons the total mole reaches 94.99%. The missing fraction in the gas composition was replaced with Louisiana Sweet crude oil.

The funnel experiment suggests that bubbles were coated with oil and an ice shell of methane hydrate. Thus, the dissolution rates accounting for the effect of surfactants were adopted with the same method described in Leonte et al.^52^ for the gaseous components^53^. The pseudo-components required to describe the oil can be found in the ADIOS oil library (https://noaa-orr-erd.github.io/ADIOS/) as “LIGHT LOUISIANNA SWEET, BP” and its incorporation method into the TAMOC is adopted from Gros, et al.^54^. Under this assumption, the volume fraction of oil at the source (compressed volumes) reaches to about 6.7%, which is smaller than the 10% limit recommended by Kvenvolden^55^ for the GoM seeps. The physical, chemical, and thermodynamic properties of bubbles were updated at each time step of the TAMOC-SBM considering the background hydrographic conditions until the bubbles either reach the surface or the gas is dissolved completely.

The TAMOC model incorporates the background currents and an averaged salinity and temperature profile for the entire depth obtained during our cruises on Jun 29 and Sept 2, 2017. To investigate the effect of variability of currents in time and space on the migration path of the bubbles with different size, we chose 50 points in time (marked with dots in Fig. 6c) to preserve the oscillatory nature of the currents particularly near the surface as much as possible while keeping the computational costs reasonable. At each time-step, bubbles with sizes ranging 1–15 mm were tracked from the source to the surface.

Since wind can affect the location of the oil once on the surface, we also show the wind data from the ERA-Interim Archive provided by the ECMWF^43^ at 10 m above the mean surface level on a 0.125° × 0.125° horizontal grid. This data is shown in Fig. 6a.

### Satellite remote sensing

At natural seeps, the oil that makes it to the sea surface spreads out into a thin layer (< 1 µm) from the oil slick origin and floats downstream with wind and surface currents^42^. SAR satellites can readily detect the oil slicks under favorable wind conditions, with speeds ranging between approximately 2–7 m/s. An oil slick will become progressively less visible and disappear over time^47^ because of weathering processes. The maximum time that oil in a slick can be detected by satellite observations is the surface residence-time^42^. A flux for the oil released from the seafloor can be estimated based on the residence time of natural oil slicks^56^. Daneshgar Asl, et al.^17^ estimate the daily flux of oil released from a seep using the equation ${Q}_{\text{SAR}}=A\zeta \left(24/{t}_{R}\right)$, where $\zeta$ is the oil film thickness.

In total, we obtained 41 SAR images collected by Sentinel-1A satellite from NASA’s Earth Science Data and Information System (ESDIS) project which provides SAR data from a variety of satellites and aircrafts through the archives maintained by the Alaska Satellite Facility. After preliminary screening of SAR images for desirable winds, the TCNNA was applied to 19 of the images to delineate the oil slicks. An empirical linear relationship for the GoM seeps, *x* = 1.2346*z* + 796.86 as suggested by MacDonald, et al.^47^, groups OSOs together when comparing among multiple images. This relation describes the lateral displacement between an OSO and its seafloor vent location, *x*, as a function of depth, *z*. For a depth of ~ 1,200 m, OSOs are clustered over a 2.3 km radius from the MegaPlume source. To obtain the oil slick area the ArcToolbox's Conversion and Spatial Statistics tools were utilized.

To estimate the surface residence-time a set of numerical simulations was implemented considering the wind and surface current conditions. The wind history was obtained from the Cross-Calibrated Multi-Platform (CCMP)^57^ project. Surface currents were obtained from the HYCOM—HYbrid Coordinate Ocean GoM 1/25° (GOM10.04) analysis experiment 32.5 Model^58^. The CCMP winds represent 10 m winds above the sea surface, with 6 h temporal resolution and are on a 0.25° × 0.25° horizontal grid. Other input parameters of the surface oil drift model are listed in Table 2. The simulations were carried out by increasing the hindcast interval in reverse time order from the image collection time in order to obtain the closest resemblance between the simulated oil pathways and the length and shape of the oil slicks observed in SAR images. The surface residence-time of the oil is estimated by using the hindcast interval that best reproduces the length and shape of the corresponding oil slick. To compare the final distribution of oil particles from the hindcast simulations against the observed oil slicks in the SAR images, we tracked the directional vectors connecting the oil slick origin location and the farthest point of each oil slick both in the model and observations.

**Table 2:** Input parameters of the surface oil drift model^17^.

Parameters	Values
Start point	Geographic location of OSO point estimated from SAR
NP^a^	100
Rs^b^	1/2 width of the oil slick at the OSO estimated from SAR
dt^c^	1
C_w_^d^	0.035
θ^e^	− 20
Start time	Estimated from surface oil drift model
End time	Date at which SAR image was collected

^a^Number of particles seeded per time step.

^b^Seeding radius (the radius within which the particles are seeded around the OSO, m).

^c^Model time step (h).

^d^Wind scaling coefficient.

^e^Wind deflection angle (degrees).

## Supplementary information

Supplementary video.

## Data Availability

Data are publicly available through the Gulf of Mexico Research Initiative Information & Data Cooperative (GRIIDC) at https://data.gulfresearchinitiative.org/data-discovery with document ids: AUV: 10.7266/N76H4FSN; Video Imaging: 10.7266/n7-3466-rn36; ASFM: 10.7266/n7-aryv-9s10; Hydrographic: 10.7266/n7-qyn1-sx24.
